# Meticulous research for design of plasmonics sensors for cancer detection and food contaminants analysis via machine learning and artificial intelligence

**DOI:** 10.1038/s41598-023-42699-6

**Published:** 2023-09-15

**Authors:** Fatemeh Jafrasteh, Ali Farmani, Javad Mohamadi

**Affiliations:** 1 Faculty of New Sciences and Technologies, Tehran University, Tehran, Iran; 2 School of Electronics Engineering, Lorestan University, Khorramabad, Lorestan Iran

**Keywords:** Cytological techniques, Physics

## Abstract

Cancer is one of the leading causes of death worldwide, making early detection and accurate diagnosis critical for effective treatment and improved patient outcomes. In recent years, machine learning (ML) has emerged as a powerful tool for cancer detection, enabling the development of innovative algorithms that can analyze vast amounts of data and provide accurate predictions. This review paper aims to provide a comprehensive overview of the various ML algorithms and techniques employed for cancer detection, highlighting recent advancements, challenges, and future directions in this field. The main challenge is finding a safe, auditable and reliable analysis method for fundamental scientific publication. Food contaminant analysis is a process of testing food products to identify and quantify the presence of harmful substances or contaminants. These substances can include bacteria, viruses, toxins, pesticides, heavy metals, allergens, and other chemical residues. Machine learning (ML) and artificial intelligence (A.I) proposed as a promising method that possesses excellent potential to extract information with high validity that may be overlooked with conventional analysis techniques and for its capability in a wide range of investigations. A.I technology used in meta-optics can develop optical devices and systems to a higher level in future. Furthermore (M.L.) and (A.I.) play key roles as a health Approach for nano materials NMs safety assessment in environment and human health research. Beside, benefits of ML in design of plasmonic sensors for different applications with improved resolution and detection are convinced.

## Introduction

The application of machine learning techniques in cancer detection has shown promising results in recent years. These approaches leverage large-scale genomic and clinical data to assist in early diagnosis, prognosis, and treatment selection. In this paper, we provide a comprehensive review of the literature on machine learning approaches for cancer detection, focusing on deep learning-based methods. We discuss various challenges and opportunities in this field, including data availability, interpretability, and generalizability^1–4^. We also explore the potential of integrating multimodal data and transfer learning techniques to improve accuracy and robustness. Furthermore, we highlight the need for standardized benchmarks, domain-specific model interpretability techniques, and ethical considerations for deploying machine learning algorithms in clinical settings^5–9^. This review aims to provide valuable insights into the current state of the art and future directions for machine learning-based cancer detection research. Recently, there has been tremendous progress in the using of artificial intelligence (AI) in different science fields ranging from engineering, optics, sensing to medicine and agriculture^10–13^. The intellectual capability of humans like social skills, creativity, abstract thinking, decision making, adapting to new environments is called general AI. AI is also defined as a study to find how machines can mimic human intelligence. Several specific AI applications can be observed in our daily life such as, product recommendation, chatbot-based customer service, photo tagging, and ext. more applications of AI in different area including design of drug, early detection of cancer and automatic and precise diagnosis are on the way. Particularly have witnessed the synergy of AI and meta-optics proposed great benefits^14–19^. Meta-optics is defined as an advanced flat optics with light-manipulation and novel function abilities. Meta-optics is an ideal choice instead of traditional optics due to its unique features like ultrathin, compact, easy to integrate and lightweight. Currently, meta-optics has been used widely in a variety of optical applications which can control the polarization, phase, amplitude, propagation direction of light and frequency^20–23^. These optical advantageous can be adjusted not only multidimensionally but also individually. Furthermore AI neural networks are considered as one of the big trend in future. There are some challenges that need to be solved namely tenability of functions, work efficiency and tenability of meta-devices. These challenges will be overcome with the improvement of fabrication technology and innovation of metasurface optics researches.

There are several machine learning techniques that can be used for cancer detection. Some of the commonly used techniques include:

1. Logistic regression: It is a statistical method that is used to predict the presence or absence of a specific disease or condition. In cancer detection, logistic regression can be used to predict the probability of a patient having cancer based on various features.
2. Support vector machines (SVM): SVM is a popular machine learning algorithm that can be used for cancer detection. It works by finding an optimal hyperplane that separates malignant and benign samples in a multi-dimensional feature space.
3. Random forest: Random Forest is an ensemble learning method that combines multiple decision trees to make predictions. It can be used for cancer detection by training the model on a set of features and their corresponding labels. The model then uses the trained decision trees to classify new samples.
4. Artificial neural networks (ANN): ANNs are computational models inspired by the structure and function of the human brain. They can be used for cancer detection by training the network on a large dataset and optimizing the weights and biases of the network to minimize the prediction error.
5. Deep learning: Deep learning is a subset of machine learning that uses artificial neural networks with multiple hidden layers. It has shown promising results in cancer detection tasks, especially with the use of convolutional neural networks (CNN) for image-based cancer detection such as mammograms or histopathology slides.
6. Genetic algorithms: Genetic algorithms are optimization algorithms inspired by the process of natural selection. They can be used in cancer detection to optimize feature selection, parameter tuning, or model selection.
7. K-nearest neighbors (KNN): KNN is a simple and intuitive machine learning algorithm. In cancer detection, it works by calculating the distances between new samples and the existing labeled samples and then assigning a class label based on the majority of the K nearest neighboring samples. It is important to note that the choice of machine learning technique depends on the specific cancer detection task, available data, and computational resources. Different techniques may yield different performance and accuracy, so it is often beneficial to experiment with multiple approaches.

**Figure 1:**
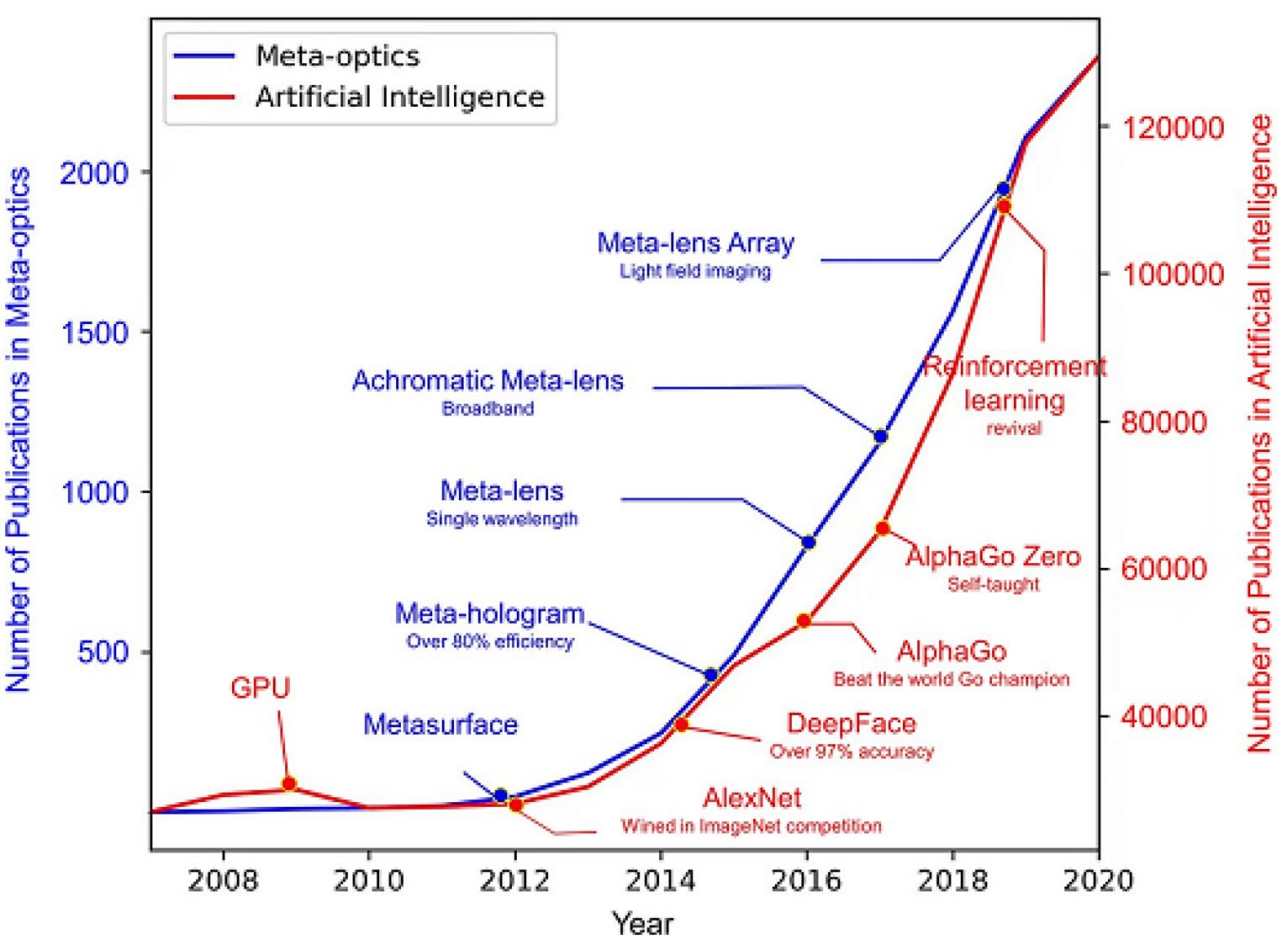
The development trend of meta-optics and AI in per year (permission from Ref.^29^).

Incorporation of AI and meta optics can break through computing speed and bottleneck of electronic computing power resulted in improvement of human civilization to a higher level. Here In Fig. 1 shows the number of publications in both meta-optics and AI has been collected^24–29^.

In the other hand AI with nanotechnology is considered the beginning of a great revolution in the medical and food applications. Food security is facing significant challenge due to the rapid increasing populations, climate change declining soil health and fertility, and increasing demand for biofuels. There is an urgent need to address these issues, therefore nanomaterials were proposed. Using nanomaterials provide a good opportunity to improve nutrient use efficiency (NUE), improving nutrient cycling and soil function by releasing the nutrient slowly for plant uptake, and using lower amount of pesticide. However the negative effects must be evaluated in parallel with the advantageous. For example, several nanomaterials NMs like TiO_2_ cause negative effects on soil community composition and modification of the bacteria community structure via negative change in denitrification enzyme activity after 90 days of exposure. More importantly delivery of NMs to soil and plants has hazardous effects to human health owing to the accumulation of active ingredient residues and NMs in specific tissues of plant. Consequently, NMs application in agriculture in a long period of time is challenging due their interaction with their surroundings^30–33^.

**Figure 2:**
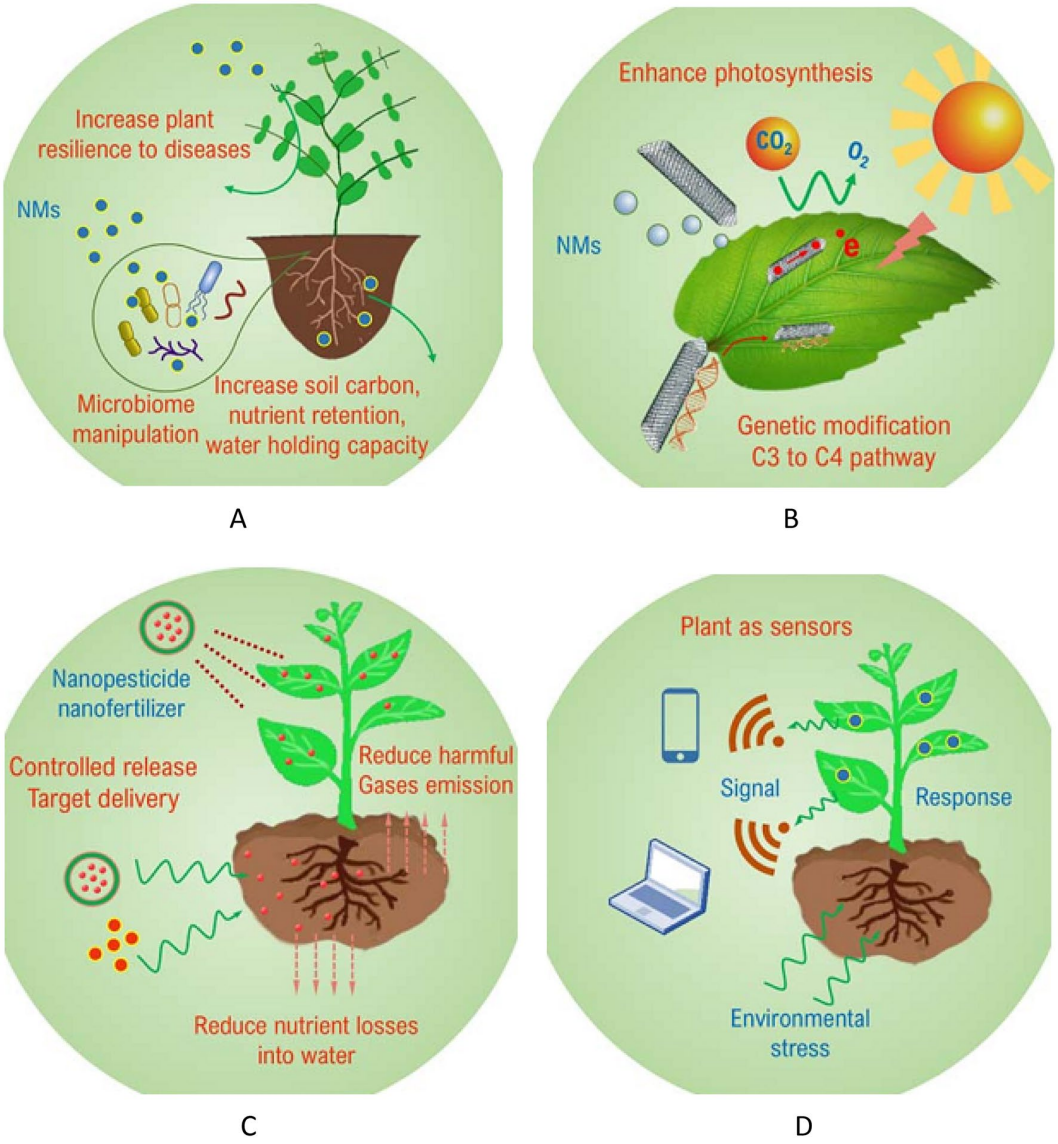
Applications of nanotechnology in agriculture (**A**) improve soil health and plant resilience, (**B**) improve production rates and crop yields, (**C**) improve resource use efficiency and reduce pollution, (**D**) plant as sensors.

Ergo a true win–win scenario that is affordable to farmers and reduces pollution from agriculture is needed. Two reliable approaches including AI and machine learning (M.L.) modeling were offered that play significant role in the progress of applying NMs in the safety way. Coupling these approaches resulted in optimization in testing and assessment of NMs process in agriculture systems. Figure 2 presents 4 different sectors where nanotechnology with AI and machine learning can modernize and revolutionize agriculture^34–39^.

- Improving soil quality and use efficiency
  Engineered NMs can improve NUE by smart controlling of release and targeted delivery for plant uptake enhancement efficiency. For instance, applying hydroxyapatite nanoneedles as urea carriers which not only lead to lower ammonia emission into the air due to the reduction of urea hydrolysis but also better yields can be achieved at 50% lower application rate. In another example, nano copper pesticides compared to the copper oxychloride demonstrate four magnitudes higher efficiency against bacterial blight on pomegranate^35–40^. More importantly nanotechnology by delivering pesticides and nutrients only at the target position leads to lower use of pesticides and fertilizers.
- Enhancement of production rates and crop yield
  One of the most promising approaches to enhance crop yield is applying plant nanobionics. A study has proved that use TiO_2_ NMs by activating the ribulose-1,5-bisphos-phate carboxylase/oxygenase carboxylation process which improve the photosynthesis efficiency as a key process in plant leaves to produce sugar from water and CO_2_ by using solar energy for plant growth. More recently, a study found that carbon dots (CDs) promote the carbonhydrate production in Arabidopsis thaliana and photosynthesis rate by increasing RuBisCO activity, leading to 20% enhance of plant yield.
- Controlling management of plant growth and soil health
  Nanotechnology are enabling to sense abiotic stressors and undesirable ambient biotic (insect damage, weed competition) resulted in better management to reduce crop loss. NMs-based sensors can not only detect plant pests or pathogens but also real time and remote detection of root exudates and metabolites to monitor crop growth. importantly corporation of NMs sensors with stimuli-responsive system (a system that deliver agrochemicals only when environmental condition changes like elevated CO_2_ or temperature, extreme pH changes, shortage of nutrients) resulted in reliable approaches to monitor resource deficiencies and plant stress.

**Figure 3:**
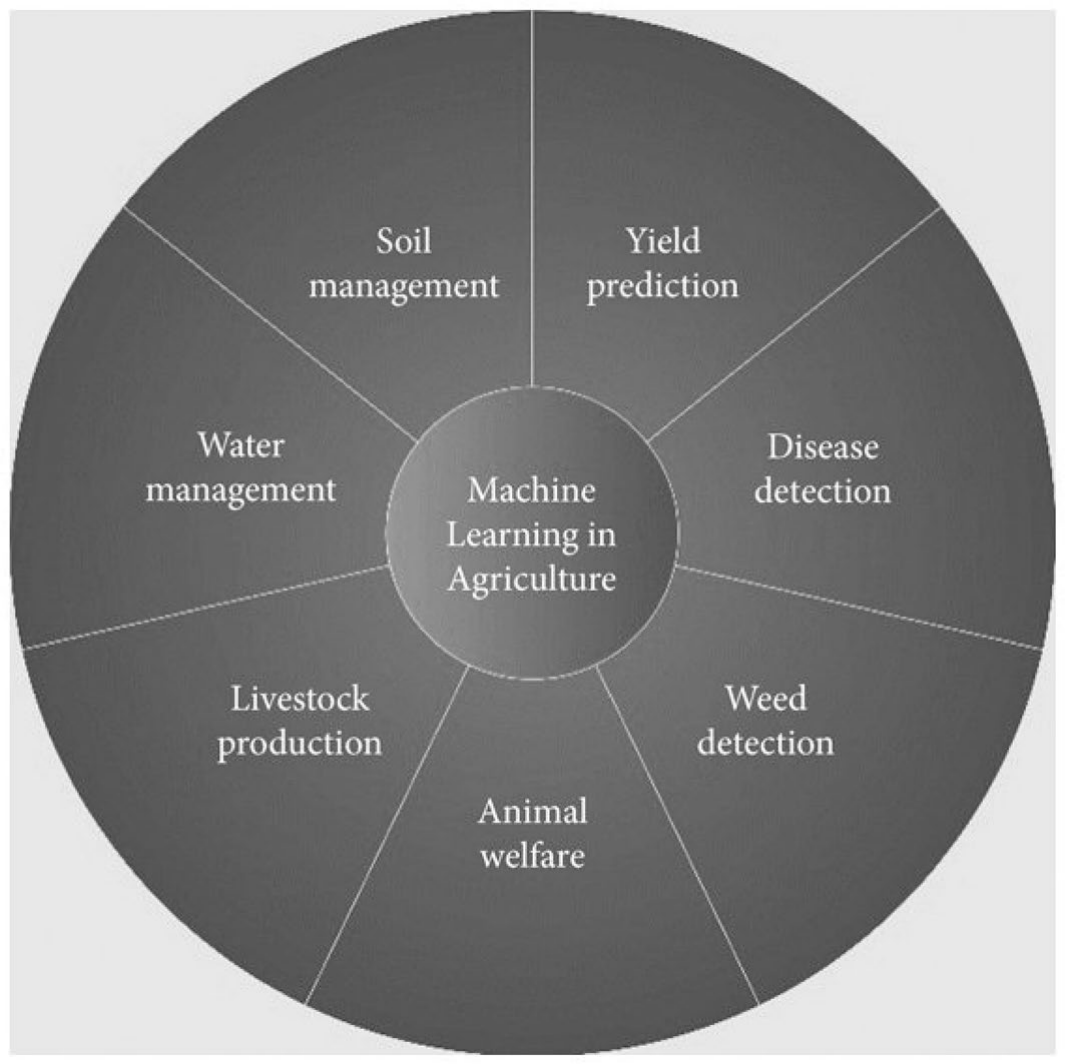
Application of machine learning in agriculture.

Most recently to achieve reliable and valid results, integrating machine learning approaches and predictive modelling for safe-by-design and risk assessment of NMs have been reported in agriculture application Due to the risks of NMs (high cost of NMs, time consuming of process, high energy input requiring to NMs synthesis, treating human health). Figure 3 presents new directions to predict faster and safer experimental approaches.

The use of machine learning in agricultural application has been classified in several areas including (a) livestock management, including livestock production and on animal welfare; (b) crop management, including disease detection, weed detection crop quality, yield prediction and species recognition; (c) soil management (prediction and discrimination of soil properties) (d) water management. Recently nanotechnology-based sensors and (AI) in biomedical and medical landscapes are in favor of numerous scientists due to the accurate monitoring of disease progress and treatment. Medical sensors can easily diagnosis and provide comprehensive information on disease symptoms from biomedical markers and fluid; however interference of irrelevant compounds in biomedical markers is a major challenge. Despite promising results, using medical sensors to detect disease is facing various limitations including: late diagnosis of disease caused treatment less efficient, low accuracy of results and limits the affordability of design of medical sensors. Hence AI-enabled medical sensors has gained an increasing attention as a system with more reliability and higher sensitivity to not only monitor disease progression and treatment process but also predict and detect specific disease.

**Figure 4:**
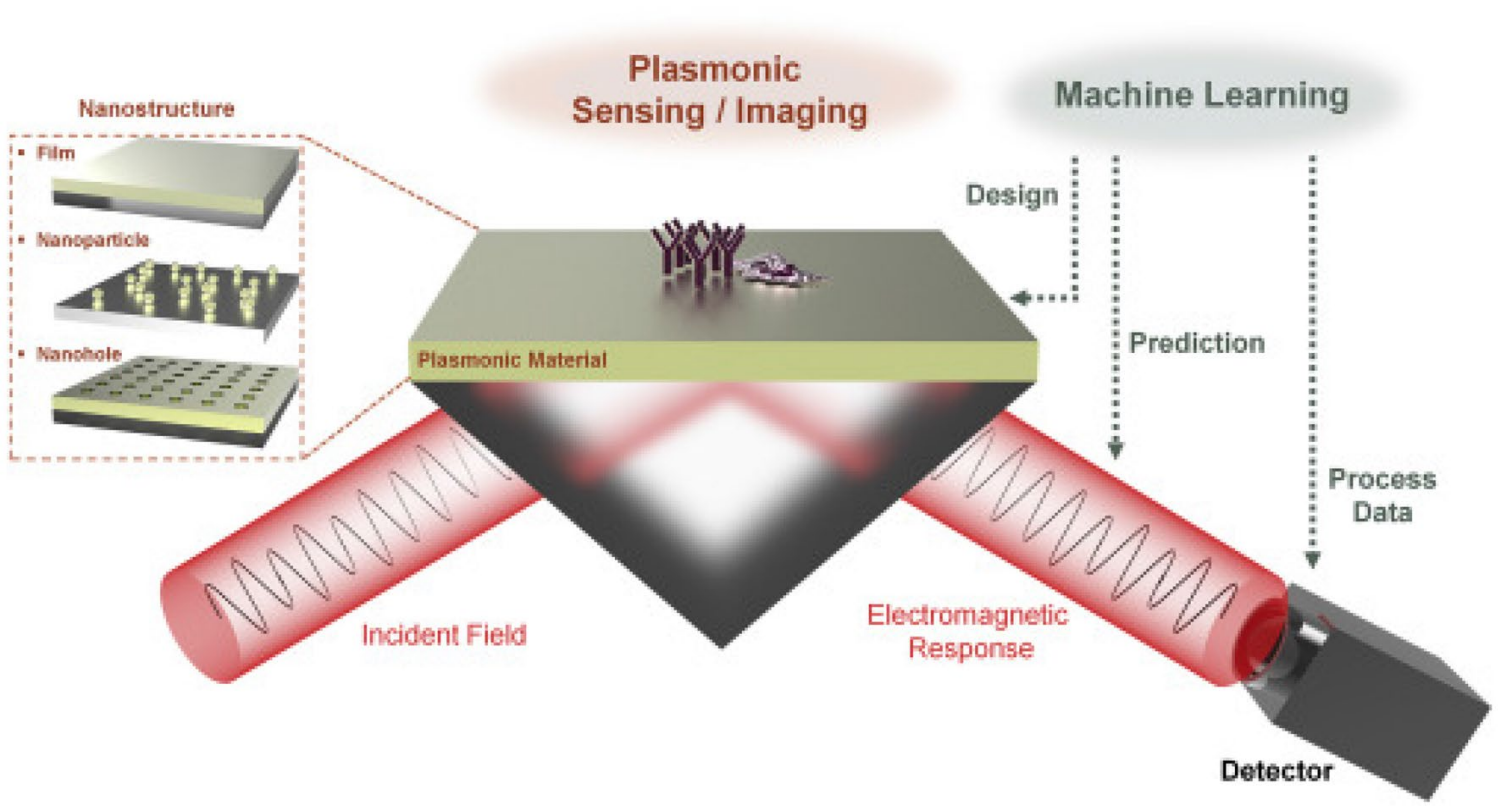
ML based data analysis for plasmonic sensors (permission from Ref.^49^).

Machine learning is artificial neural networks (ANNs) that can be divided into two groups: regression and discriminative. Generative models are applied for creation of new data points whereas discriminative models are used for regression or classification. Generative adversarial networks (GANs) and variable autoencoders (VAEs) are generative models. Discriminative models like multilayer perceptron (MLP) and convolutional neural networks (CNNs). Furthermore tremendous interest has been drawn to use ML for creation smart SPR sensors. The surface plasmon resonance (SPR) sensors are used widely in bio-sensing applications. SPR is an attractive tool due to its ability to monitor molecular interactions in quick response^40–47^. From Ref.^48^, Analysis of data from SPR sensors-based ML have been is shown Fig. 4.

**Figure 5:**
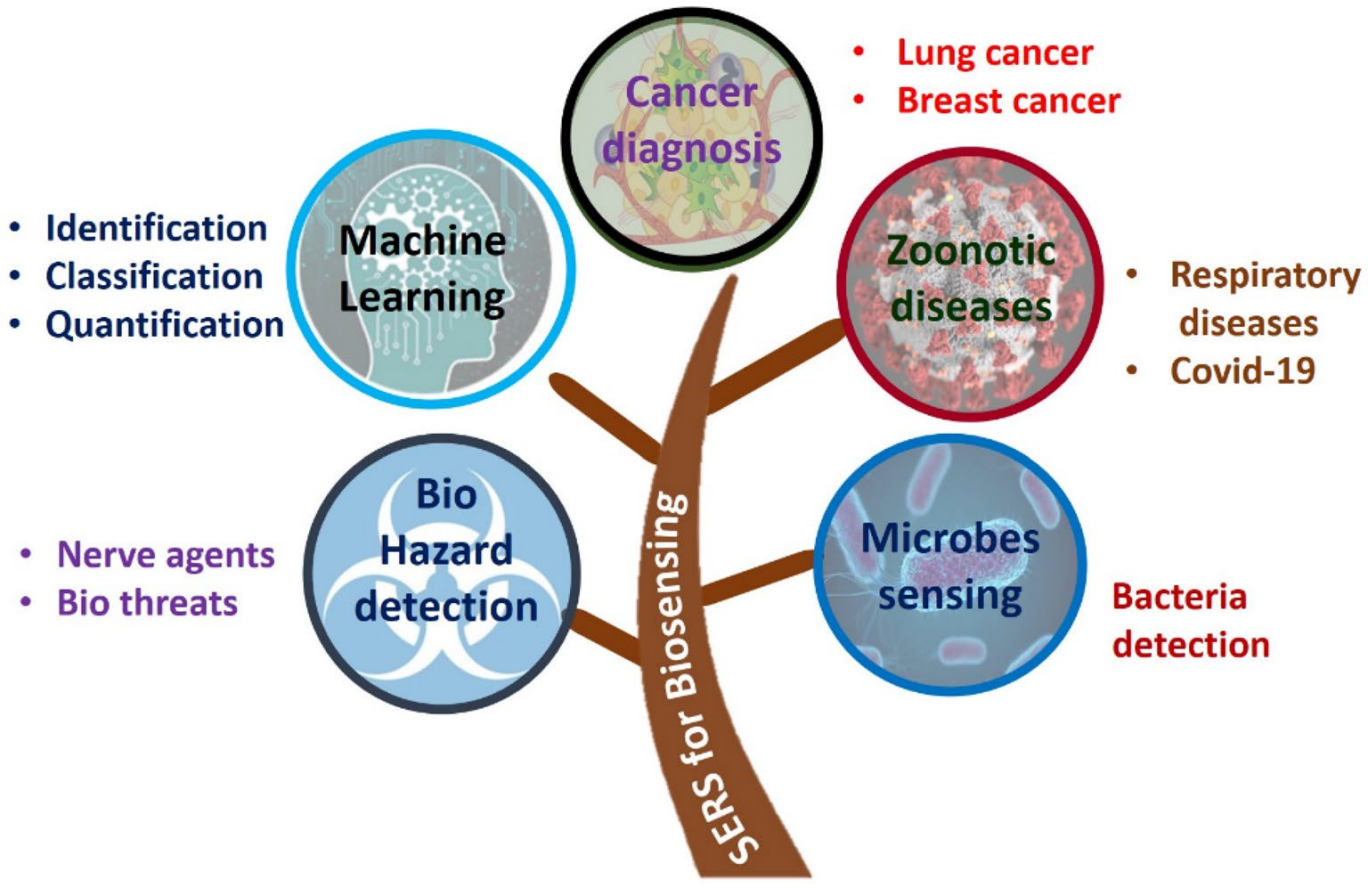
Applications of ML using SERS in different field of biosensing.

Furthermore, these techniques have been used widely in cancer disease modeling for stratification and prediction, assessment of the degree of DNA damage, determination of virus size. Specifically, in the field of biology applications, ML models that were used in SERS enhance the usability as shown in Fig. 5.

### Challenges in deep learning-based cancer detection

1. Lack of labeled data: deep learning models require a large amount of accurately labeled data in order to be trained effectively. However, obtaining labeled data for cancer detection can be challenging due to factors such as patient privacy concerns, limited access to high-quality medical images, and the expertise required to accurately annotate the data.
2. Interpreting complex results: deep learning models often provide highly accurate predictions, but understanding how and why the model arrived at a particular decision can be difficult. This lack of interpretability makes it challenging for physicians to fully trust and act upon the model's output, especially in critical decisions related to cancer diagnosis and treatment.
3. Class imbalance: cancer datasets are often imbalanced, with a smaller number of positive cases (i.e., cancer instances) compared to negative cases (i.e., non-cancer instances). This imbalance can lead to biased models that perform well on majority class prediction but struggle with minority class prediction. Overcoming class imbalance requires careful data preprocessing techniques or algorithm modifications.
4. Generalization to different populations: deep learning models trained on one population may not generalize well to different populations due to differences in demographics, genetics, and other factors. This creates a challenge for deep learning-based cancer detection models to provide accurate predictions across diverse patient populations.
5. Data quality and noise: medical imaging data, such as mammograms or CT scans, can be noisy and have artifacts that can adversely affect the performance of deep learning models. Ensuring the quality of the data and reducing noise is important to avoid false positive or false negative predictions.
6. Deployment in clinical settings: translating deep learning models from research settings to real-world clinical practice presents its own challenges. Integrating the models into existing healthcare systems, ensuring their seamless integration with electronic health record systems, and addressing regulatory compliance and legal considerations can be complex.
7. Ethical considerations: deep learning-based cancer detection raises important ethical concerns, such as ensuring the privacy and security of patient data, addressing biases in the models, and minimizing the potential impact of incorrect predictions on patient outcomes. These ethical considerations need to be carefully addressed to build trust and ensure responsible deployment of the technology.

Overall, addressing these challenges is crucial to successfully harness the potential of deep learning in cancer detection and improve patient care.

### Plasmonics sensor for cancer detection

**Figure 6:**
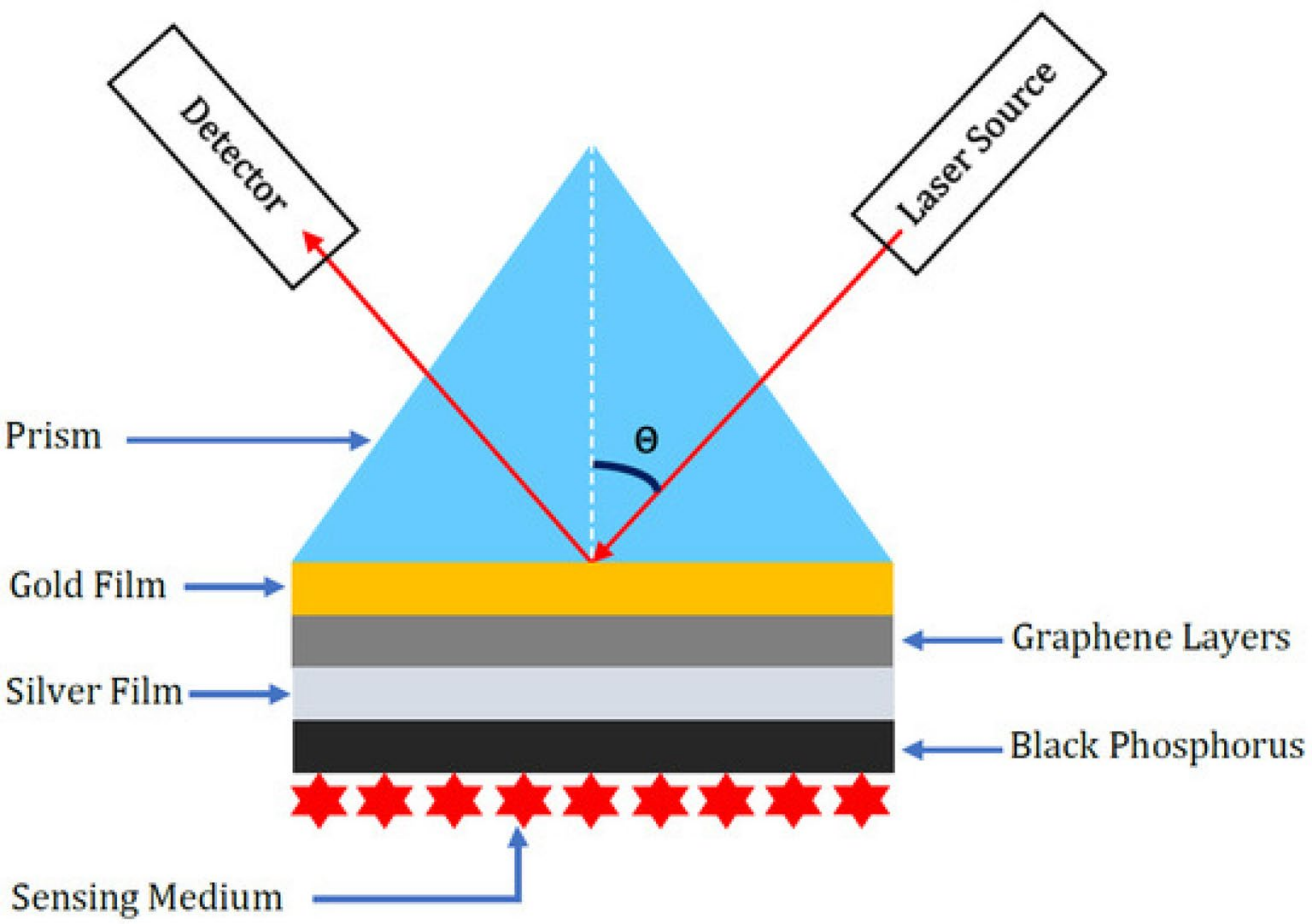
plasmonics sensor for cancer detection (^52^, our recent work).

One-way plasmonics sensors can be used for cancer detection is through surface-enhanced Raman spectroscopy (SERS). SERS is a powerful technique that can identify specific molecular compositions based on their unique vibrational spectra. By integrating plasmonic nanostructures such as gold or silver nanoparticles with biological samples, the sensitivity of SERS can be significantly increased^45–53^ (see Fig. 6).

In cancer detection, plasmonic SERS sensors can be used to identify cancer-specific biomarkers present in body fluids, such as blood or urine. These sensors can be designed to selectively bind to cancer biomarkers, enabling their detection at extremely low concentrations. By analyzing the Raman scattering of the bound biomarkers, plasmonic SERS sensors can provide accurate and rapid cancer diagnostic information. Furthermore, plasmonic sensors can also be used for the detection of circulating tumor cells (CTCs), which are cancer cells that have detached from the primary tumor and entered the bloodstream. Plasmonic sensors can be designed to capture and detect CTCs based on their unique molecular and physical properties. This enables non-invasive monitoring of cancer progression and response to treatment. Overall, plasmonics sensors offer a promising technology for cancer detection. Their high sensitivity, specificity, and ability to analyze complex biological samples make them a valuable tool in the fight against cancer. However, further research and development are needed to optimize the performance and translation of these sensors into clinical applications^53–55^.

## Conclusion

In this perspective the necessary of (M.L.) and (A.I) in data processing were discussed. Divers M.l based methods like classification, regression and dimension reduction and their applications have been studied. Newly promising AI and meta-optics applications in design of intelligent optical devices have been reported. Noticeably A.I and machine learning can progress nano enabled agriculture result in reduction of environmental pollution and negative human health impacts. (A.I) and (M.I.) could be predicted the behavior of NMs that facilitate the design of NMs to enhance crop yield and NUE. ML-based approaches provided significant features from complex data from plasmonic biosensors. The results provided accurate and reliable estimates of sample analysis with low concentration and quick time. Looking further ahead AI and ML will bring unlimited possibilities for data gathering with high accuracy in the next generation of different sensor devices such as medical sensors and biosensor.

## Data Availability

All data included in this paper are available upon request by contact with the contact corresponding author.
